# Increase in War Office Capitation Grant

**Published:** 1918-02-23

**Authors:** J. Courtney Buchanan, Conrad W. Thies

**Affiliations:** Hon. Secretarie. 14 Victoria Street, Westminster, S.W. 1.; Hon. Secretarie. 14 Victoria Street, Westminster, S.W. 1.


*To the Editor of* The Hospital.

### Increase in War Office Capitation Grant.

*To the Editor of* The Hospital.

Sir,—We now have pleasure to forward you a copy of Army Council Instruction No. 144 of 1918, dealing with this subject. (See page 446.)

As a result of the conference at the Westminster Hospital on October 19, 1917, a meeting of the Council was held, and a deputation was appointed to meet the representatives of the War Office.

The deputation, representing both London and provincial hospitals, waited upon Sir Charles Harris, Financial Secretary to the War Office, on November 16, 1917. As a result of the discussion which then took place it was decided that the consideration of certain details should be referred to a Joint Committee of the British Hospitals Association and of the War Office.

The Joint Committee met on several occasions, representative hospitals in various parts of the country were visited, and a comprehensive report was drawn up.

On February 1, 1918, Sir Charles Harris again received the deputation; and, as will be seen from the Army Council Instruction enclosed, a substantial increase in the capitation grant was conceded, and a grant of 6d. per diem for each unoccupied bed was authorised—both taking effect as from October 1, 1917.

Yours faithfully,
J. Courtney Buchanan,
Conrad W. Thies,
Hon. Secretaries.

14 Victoria Street,
Westminster, S.W. 1.
February 14, 1918.

